# Strengthening health research capacity for postgraduate trainees: an indigenous realist evaluation of the ‘African Research Initiative for Scientific Excellence’ programme

**DOI:** 10.1093/heapol/czaf055

**Published:** 2025-08-20

**Authors:** Meshack Nzesei Mutua, Catherine Nakidde, Ferdinand C Mukumbang

**Affiliations:** Department of Organisational Psychology, Leslie Commerce Building, Upper Campus, University of Cape Town, Rondebosch, Cape Town 7701, South Africa; Research Department of Medical Education, UCL Medical School, University College London, Gower Street, London WC1E 6BT, United Kingdom; Department of Global Health, School of Public Health, University of Washington, Hans Rosling Center, 3980 15th Ave NE, Seattle, WA 98105, United States

**Keywords:** Indigenous realist evaluation, research capacity strengthening, (post)graduate training

## Abstract

International research partnerships are crucial to strengthening research capacity (RCS) efforts. However, little is known about how such partnerships work to enhance the capacity of postgraduate trainees. We applied an Indigenous realist evaluation (RE) approach to examine how the ‘African Research Initiative for Scientific Excellence’ (ARISE) programme works to strengthen the capacity for trainees. The Indigenous RE integrates critical and scientific realism paradigms with the Postcolonial Indigenous paradigm, focusing strongly on power, relationality, and decolonization. We used a multi-case study design to investigate two cases of innovation- and laboratory-based research projects led by African principal investigators (PIs). We conducted realist-informed interviews and observations with PIs, interviews with collaborators and partners, and storytelling with students. Realist thematic analysis helped to identify context, intervention, mechanism, and outcomes (CIMO). Deductive, inductive, abductive, and retroductive reasoning were applied to generate programme theories through an iterative and rigorous theory-building process. Findings show that trainees who are committed and self-driven, based in a research-intensive university that provides complementary opportunities and where there is demand for multidisciplinary research, will improve their skills, secure additional funding, and transition from master’s to PhD programmes. This is because the RCS resources would inspire, challenge, empower, activate a sense of agency, and provide the trainees with eye-opening experiences. However, trainees would secure jobs outside Africa (brain drain) if career opportunities in specialized fields are limited locally. If trainees are junior faculty staff and fully funded, and their university provides protected time, RCS resources would inspire, motivate, and empower them, resulting in increased research outputs and career growth. RCS efforts targeting (post)graduate trainees need to consider ‘inter alia’ the university contexts (e.g. availability of complementary resources and protected time), the individual traits and readiness for postgraduate training, and the broader ecosystem, which determines if the trainees’ skills benefit Africa’s research and development.

Key messages

- International research partnerships are pivotal in delivering health research capacity-strengthening support for masters/PhD trainees. Evidence on how, why, and under what circumstances such complex initiatives work across contexts is not well developed.
- Using an Indigenous-inspired realist evaluation, we have established that empowerment, inspiration, eye-opening experiences, sense of agency, motivation, and commitment are key mechanisms needed to generate improved research capacity outcomes.
- While health research capacity strengthening aims to enhance local capacity, the empowerment of the (post)graduate trainees might generate undesired outcomes as they seek career opportunities outside the African continent, thus depriving the continent of the much-needed research capacity and consequently creating more inequity between the North and the South divides. Research capacity strengthening efforts should go beyond supporting individuals to improving institutional and systemic capacities, thereby providing a conducive research environment where both research and researchers can thrive.

## Introduction

Developing a critical mass of researchers committed to research and development (R&D) is central to addressing the world’s development challenges (Rivero 2024). African countries are significantly underserved compared to the Global North countries (Ayan *et al.* 2023, Rivero 2024). To address this concern in global health, research capacity strengthening (RCS) has been widely adopted as a strategy to counter health disparities (Nuyens 2005, Irfan *et al.* 2022, Adam *et al.* 2023). The ESSENCE on Health Research (2023) has defined RCS as ‘enhancing the capacity of individuals and organizations to conduct, manage, share and apply research, while enabling national and sub-national research systems to effectively support both research and the linkages between research and practice’ (p. 3).

Literature shows that RCS occurs at three different levels, including individual, institutional and national/international and, sometimes, concurrently cutting across the three levels (Lansang and Dennis 2004, Bates 2015). At the individual level, RCS efforts may target researchers, technical specialists, research managers or masters/PhD students levels, at the institutional level, they will focus on strengthening for instance research governance, upgrading research infrastructure and equipment, income streams, ethical approval processes, library/IT support, communication services and at the (inter)national level, they will target policymakers, donors, professional associations, research networks, institutional partnerships, state, and corporate stakeholders (ESSENCE on Health Research 2023). While RCS initiatives are mostly embedded within research projects, say, research on malaria, mental health or HIV, others might solely involve the implementation of RCS activities/objectives (Pulford *et al.* 2021).

Despite the distinction, RCS efforts overlap across levels, making them complex and heterogeneous (Ager and Zarowsky 2015). Even when an RCS initiative is aimed at strengthening capacity at the individual level, multiple contextual factors at micro (individual), meso (institutional), and macro (system) levels will likely moderate the research capacity outcomes in ways that cannot be anticipated (Ager and Zarowsky 2015, Pulford *et al.* 2021). For instance, most sub-Saharan African universities have underdeveloped and struggling research functions that create constraining environments that make postgraduate training complex and challenging (Mentz-Coetzee *et al.* 2020). Despite this complexity, evaluation of such programmes does not engage with the complexity of such initiatives to establish what makes them work, why, and how across varied contexts (Pulford *et al.* 2021).

International research partnerships have been central to RCS (Nchinda 2002, Barrett *et al.* 2011, Dean *et al.* 2015, Varshney *et al.* 2016). Partnerships involve two or more entities working together/collaborating towards a common goal (Plamondon *et al.* 2021). Geographically, international partnerships have been forged between the global North-South, South-South, and North-North (Blicharska *et al.* 2021), and international research partnerships have sought to foster interdisciplinary research by leveraging a combination of skills, expertise, resources, and facilities to address complex research questions impactfully (Carter 2023). Partnerships between high-income countries (or the global North) and low-income countries (or the global South) involve RCS efforts in the South (Kellerman *et al.* 2012, Atkins *et al.* 2016). While some North-South partnerships have been critical in training researchers and strengthening research leadership in LMICs, Horwood *et al.* (2021) argue that others have been criticized for imbalanced power dynamics with the global North partner(s) controlling what happens in the LMIC contexts. Many international research partnerships are working relationships between unequal partners, with the inevitable emergence of asymmetric and unbalanced power relations (Zingerli 2010). How the international research partnership is construed should thus be considered as it determines its design, ethics, and impacts (Perry *et al.* 2022).

Given the centrality of international research partnerships in delivering health RCS efforts, which is geared towards improving research capacity in the Global South (Morel *et al.* 2018, Rivero 2024), there is an increasing need to provide policy-relevant evidence on what, why, and how the partnerships work and what under what conditions (Marjanovic *et al.* 2017). Using the ‘African Research Initiative for Scientific Excellence’ (ARISE) programme as a real-world case of an international research partnership, this paper applies an Indigenous realist evaluation approach to examine how the programme works, why, for whom, and under what circumstances to strengthen research capacity for postgraduate trainees in a middle-income country. The Indigenous lens was deemed critical in exploring and understanding power dynamics in the ARISE partnership, centring the participants’ voices in building, testing, and refining the programme theory. In our recently published paper, we report on the equitable nature of the ARISE partnership, delineating the power dynamics between the funding and implementing partners, as well as the project-level stakeholders and the mechanisms activated to achieve equity (Mutua *et al.* 2025). The design of the ARISE initiative followed a Bottom-Up approach where the African actors were allowed to define their research priorities, design their research initiatives in alignment with their local/national priorities and, importantly, enjoyed autonomy in implementing their research initiatives, something that was crucial in fostering equity as voiced by the African stakeholders (Mutua *et al.* 2025). This study asks: how and why does the ARISE—an initiative founded on an equitable partnership—work to strengthen research capacity for postgraduate trainees based in African universities characterized by varied contextual conditions? And what mechanisms are activated by the ARISE across the targeted university contexts to generate desired or undesired, expected or unexpected research capacity outcomes for the postgraduate trainees?

### The ‘African Research Initiative for Scientific Excellence’ programme

The ARISE programme, a 5-year (2022–2026) initiative, is a global North-South partnership with the European Commission as the funding partner and the African Academy of Sciences and the African Union as the implementing partners. One of the objectives of the programme is to strengthen the capacities of the emerging African research leaders committed to a research and teaching career in Africa. The programme specifically targets early-career researchers (PIs) who have demonstrable potential to become research leaders in Africa. The researchers are funded to implement research projects that address specific research questions (e.g. related to health, climate change, etc.) and local development challenges in their countries. To implement their research projects, the researchers are expected to establish research collaborations, leverage their collaborators’ research equipment and expertise and, importantly, recruit, train, and supervise master’s and PhD trainees.

### The indigenous realist evaluation

An indigenous realist evaluation approach takes a multi-paradigmatic stance, including realist and postcolonial indigenous paradigms (Mutua and Nakidde 2024). While the realist approach by Pawson and Tilley (1997) does not have an emancipatory dimension, the Indigenous lens brings in the emancipatory power and intentional pursuit of epistemic justice by interrogating power structures and dynamics within an intervention (Mutua *et al.* 2025). Adding the postcolonial Indigenous lens to the realist evaluation approach, which is anchored in critical and scientific realism principles, also encapsulates the emancipatory potential of realist evaluation from a critical realist perspective (Mukumbang *et al.* 2023).

**Figure 1.**
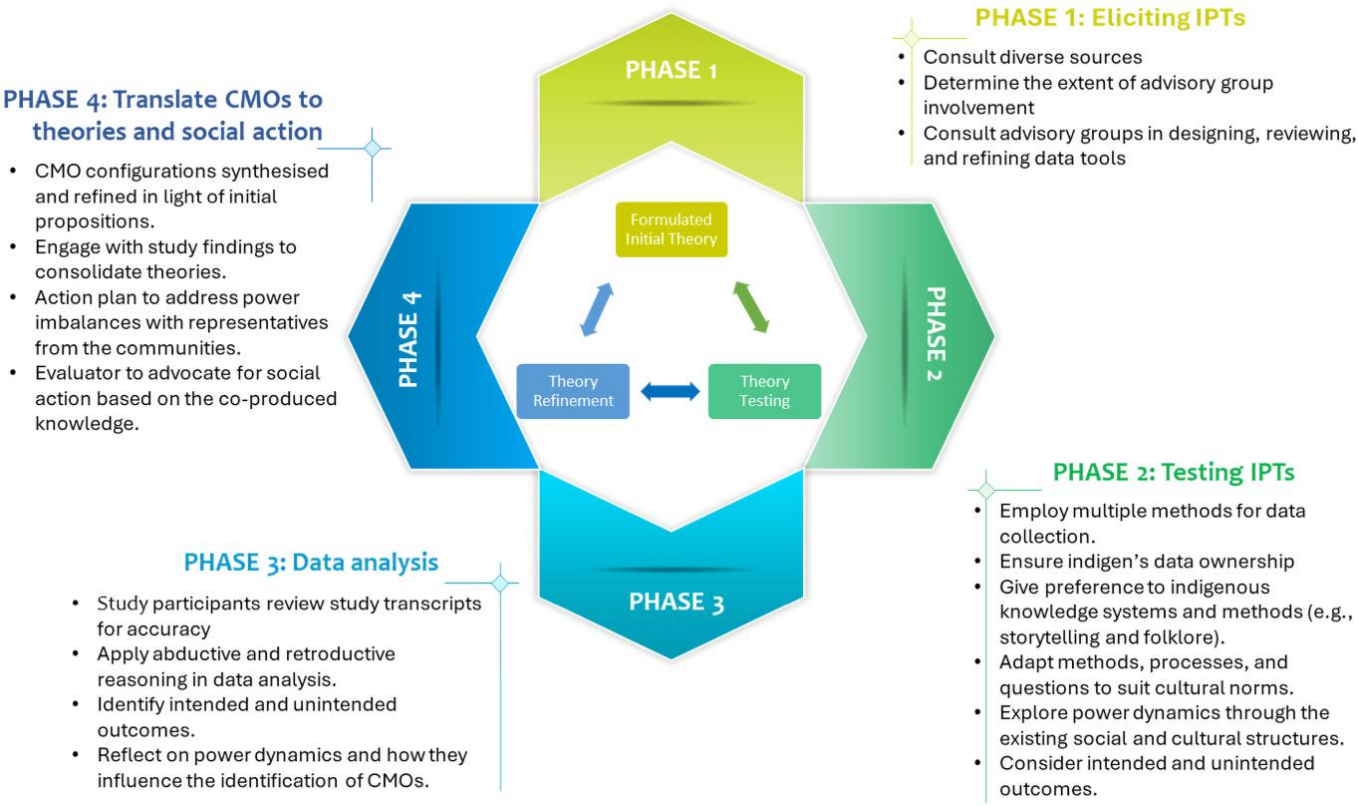
An indigenous realist evaluation cycle adapted from Mutua et al. (2025).

When researching Indigenous or formerly colonized societies, the postcolonial Indigenous paradigm focuses on the postcolonial structures, ideologies, and power dynamics (Chilisa 2012). The Indigenous-informed realist evaluation approach builds on Tinsley’s (2022) postcolonial critical realism perspective, which emphasizes the need to interrogate the (post)colonial structures and power dynamics, how they manifest in the ‘actual, real, and empirical’ domains and shape, influence, and affect people’s experiences of social interventions Conducting a realist informed Indigenous revaluation entails that the evaluation adheres to or respects the Indigenous research principles—see Fig. 1 below. By doing so, the evaluation would not only address the realist question of what works, how, why, for whom, and under what circumstances (Pawson and Tilley 1997) but also goes further to ensure that the voices of the varied participants are given primacy, the participants are engaged throughout the process. The evaluation adds value by seeking to lay bare and address power imbalances and epistemic injustice.

The Indigenous realist evaluation fuses the realist evaluation approach with the Indigenous research principles described by Chilisa and Mertens (2021) namely ‘relationality, responsibility, reverence, reciprocity, respectful representation, reflexivity, responsivity, rights and regulations, and decolonization’. Mutua and Nakidde (2024) have suggested how the two can be fused and operationalized in a health research capacity-strengthening programme. The goal remains to formulate mechanism-based theories undergirded by participants’ experiences of an intervention to explain how and why social interventions work (Pawson *et al.* 2005). However, since Indigenous realist evaluators will engage and work with communities that are disenfranchised and disempowered by (post)colonial structures and ideologies, the evaluations should then be geared towards transformative change (Mutua *et al.* 2025). The evaluation seeks to address power imbalances and inequities where and how they exist (contexts) and how this influences/shapes people’s reasoning, concerns, perceptions, and agency (mechanism) to generate outcomes. Therefore, the evaluator takes an active role in identifying and calling out power imbalances and inequities (stemming from colonial structures and ideologies, or otherwise) and forging effective relationships with programme actors to ensure appropriate actions are taken (Mutua *et al.* 2025).

### Student training and supervision—initial programme theory

All the ARISE PIs were expected and encouraged to recruit, train, and supervise up to four master’s and two PhD students over their project duration. The students were to be embedded within the ARISE research projects and benefit from the wide range of research support, infrastructure, and expertise provided by the PIs and their collaborators. By training and supervising students, the ARISE PIs would have the opportunity to test their research leadership skills and pass on the skills acquired through their participation in research capacity-strengthening activities. To assess the contribution of the ARISE programme to RCS of the postgraduate trainees, an initial programme theory (IPT) was first elicited.

Firstly, we reviewed six of the ARISE programme documents, including the funding proposal, the programme description of action, the M&E framework, and the grantees’ progress reports to generate a rough IPT. Since the ARISE documents did not explicitly highlight the contexts and mechanism, we gleaned and extracted a few relevant context and outcome elements—see Supplementary File S1. Secondly, we revised the IPT by conducting three focus group discussions with the project-level participants and three interviews with the programme-level partners. The realist interviewing technique was employed, allowing participants to comment on the IPT. Supplementary File S1 presents the context, mechanism, and outcome elements and codes from the FGD and interview data, along with their application in modifying the draft IPT. Finally, we refined the IPT further by reviewing six published papers describing health RCS. Although the literature review did not yield new insights, it was crucial in confirming the plausibility of the IPT as framed. For instance, ‘trainees transition to seasoned/full-time health researchers in Africa’ and ‘research facilities, supervision, research skills training, student welfare, and PhD office space’ were supported by literature as potential outcomes and contexts, respectively. The refined IPT was framed as shown in Box 1 below.

Box 1. **Initial programme theory**

**IF** the PI has sufficient supervisory capacity (C1), has established intra-African research collaborations (C2), is based in a research institution that has adequate government funding to support the training of masters/PhD students (C3), and the trainees have financial support and access to a wide range of research expertise and equipment (I),

**THEN** the masters/PhD trainees will transition into seasoned health researchers in Africa (O),

**BECAUSE** they will acquire the right knowledge and perceived support (M).

## Methods

A multi-case realist evaluation design was employed. Case study design is useful in analysing, refining, and presenting programme theories specific to each Case (Gilmore *et al.* 2019, Knap *et al.* 2023). Although the wider realist study considered three case studies labelled as Case A, B, and C (see Supplementary Figure S1), only two cases (Cases A and C) recruited students in their capacity-strengthening component and were included in our analysis. Each case represented an ARISE research project based in an African university. The selection of cases considered the host university's size, the country's economic status, and the nature of the research project—see Table 1.

**Table 1.** Characteristics of the cases.

Case	Nature of research project	Host institution	Country’s economy status
Case A	Innovation-based research	Research-intensive public university	Upper middle-income country
Case C	Laboratory-based research	Research-intensive public university	Low-income country

The case studies were conducted sequentially, allowing insights from one case study to be shared with the next. Both within-case and cross-case analyses were carried out to generate case-specific theories and an overarching theoretical framework (Gilmore *et al.* 2019, Mukumbang 2021).

### Data collection and analysis

Realist interviews and storytelling sessions were employed. The choice of methods was informed by the methods-neutral nature of realist research (Pawson 2006, 2013) and the need to integrate Indigenous methods such as storytelling (Chilisa 2015). Although the ARISE researchers (PIs) were expected and encouraged to recruit, train, and supervise up to four master’s and two PhD students over their project’s duration, different contextual challenges affected the recruitment and training. Purposive sampling allowed the selection of participants who had a meaningful experience with the ARISE programme and could provide useful information needed to test the IPT on student training and supervision. The realist study targeted diverse programme participants (i.e. PIs, collaborators, PIs’ mentors or supervisors, research support staff and finance and grants personnel, masters and PhD students, and the programme staff and partners) across the cases. Table 2 summarizes the participants and the data collection method used.

**Table 2.** Participants and data collection methods.

Participant category/data collection method	Number
Interviews and participant observations with PIs	2
Interviews with research collaborators	6
Storytelling with master’s and PhD trainees	8
Interviews with partners	3
Total number of participants	19

The data collection aimed to test the IPTs and validate or refute them based on the participants’ experiences with the ‘student training and supervision’ component of the ARISE programme. The refined programme theories should articulate the elements of contexts that trigger specific motivations, incentives, reasoning, or resource aspects of mechanisms to generate positive or negative, intended or unintended outcomes (Dalkin *et al.* 2015). The interview questions were framed around contexts, mechanisms, and outcomes, and probing questions were appropriately used to uncover deeper evidence for theory development. The realist interviewing technique was applied to gather ontologically deep evidence on context and mechanism (Westhorp and Manzano 2017, Verkooijen *et al.* 2020). The idea was, in line with Westhorp and Manzano (2017) suggestion, to elicit the participants’ views and experience by posing our theorized inner workings of the ARISE programme. While the art of realist interviews is considerably well developed (Manzano 2016, Westhorp and Manzano 2017, Mukumbang *et al.* 2020, Verkooijen *et al.* 2020, Brönnimann 2022, Strachan *et al.* 2022), the storytelling approach was adapted in our study to ensure that meaningful and latent mechanism were uncovered (Mutua 2025).

Participants were allowed to first share their experiences with the ARISE programme (narrative stage), mainly reporting on context and to some extent outcomes. Then, realist follow-up interviews were conducted to uncover ontologically deeper mechanisms, with the interviewer posing the IPT to the participants so they could comment on the extent to which the theory squared with their experiences (Mutua 2025). Some of the probing follow-up realist questions and excerpts of participants’ responses have been shared by Mutua (2025). The evidence collected was examined to determine if and to what extent the participants’ experiences squared with the ‘student training and supervision’ IPT. Given the sequential case study implementation design and in line with the Indigenous ‘responsivity’ principle, the interview questions and process in Case C were adapted based on the experiences in Case A. For instance, questions were added to the interview guide to probe and distinguish the availability of ‘complementary training resources provided by the university’ and those provided by the ARISE programme.

Before the data were analysed, and in line with the Indigenous ‘relationality’ principle, participants were given the opportunity to review their transcripts and edit as necessary. By doing so, the participants would have control over what was written, and their voices would consequently be central to the CMO configuration process (Mutua *et al.* 2025). Some participants took the opportunity to clarify certain aspects of their accounts and provide additional details about changes that had occurred since the interview (Mutua *et al.* 2025). This also adhered to the Indigenous principle of ‘rights and regulation’, which acknowledges the participants’ rights to data ownership.

A realist approach to thematic analysis (Wiltshire and Ronkainen 2021) was applied. This helped to identify experiential, inferential, and dispositional themes from the data by applying deductive, inductive, abductive, and retroductive reasoning (Mukumbang 2021, Mukumbang *et al.* 2021). We applied both deductive reasoning (guided by the IPT) and inductive reasoning (data-driven) to code each transcript to identify experiential themes that describe the participants’ thinking, motivation, and feelings about the (post)graduate training and supervision within the ARISE programme and, how and why the programme improved research capacity for the trainees. The inductive reasoning, for instance, allowed us to understand the data as it is without fitting it into the IPT. The inductive approach, thus, created the opportunity for our preconceptions to be challenged by the new insights emerging from the data, thereby enabling us to identify all possible CIMO configurations from the data (Mutua *et al.* 2025). Using deductive reasoning only can be disadvantageous, as it may encourage the evaluator to find what they are looking for, thereby introducing the risk of confirmation bias. Although the goal of realist evaluation is to test, refine or refute the IPT, using an IPT to guide coding, according to Meyer and Lunnay (2013), can make the researcher impose their theory onto the data, thus overlooking alternative ways of making sense of the data. To this end, the deductive approach was primarily used to formulate realist interview questions that will allow us to test the IPT. The use of inductive reasoning helped to avoid this confirmation bias, hence making the analysis process robust.

Our coding and experiential thematic analysis followed the dyadic (e.g. mechanism-outcome) and triadic (context-mechanism-outcome) approach proposed by Jackson and Kolla (2012). Following the experiential thematic analysis, we applied a hybrid inductive and abductive reasoning to link the experiential themes to generate tentative CIMOCs that explain how the programme works to strengthen the capacity of the trainees across the two university contexts. Realist researchers have applied different variations of context-mechanism-outcome frameworks to improve the clarity of generative causality (Rees *et al.* 2025), and in this case, we included the intervention (I) in the configuration [CIMO] to demonstrate the kind of resources, opportunities, or constraints provided to the programme recipients. The linked constructs were then aggregated (in an inferential analysis step) to generate overarching CIMOCs. Lastly, through a discursive session, we examined the inferential CIMO links to generate a causal loop diagram that depicts the causal mechanisms and the contextual conditions for the trainees’ research capacity outcomes to be generated. We focused on the voices and experiences of the wide range of programme participants, which aligns with the Indigenous ‘relationality’ principle—delineating the experiences of the (post)graduate trainees and the views of the other participants. For each programme theory, we constructed ‘if…then…because… statements’ representing ‘What works, for whom, how, and why, and under what conditions’. The statements capture the essence of doing a realist evaluation—to unearth what works, for whom, why, and under what conditions and which can facilitate translation of evidence to practice (Mukumbang *et al.* 2025). Following the formulation of the programme theories, ARISE participants were involved in validation and dissemination workshops, where the programme theories were presented and the potential next course of action discussed, in line with the principles of ‘rights and regulations’, as well as ‘respectful representation’. During the workshops, participants had the opportunity to reflect on the evidence and propose recommendations that their host institutions (universities), the ARISE programme staff, and partners could implement to optimize the programme’s impact.

Since this study was conducted as part of a PhD research project (by the lead author), the Indigenous ‘reflexivity’ principle played a pivotal role in clarifying the researchers’ positionality. As an African PhD student himself and exploring the subject of ‘masters and PhD training’ in Africa, the lead author assumed an insider’s perspective on the subject (as he identified with most of the contextual conditions reported by the ARISE trainees) and, therefore, the researcher’s positionality and how it influenced data collection and analysis were subjects of discussion among the co-authors. As a postgraduate student in a Global North university and employing a realist methodology in her PhD research, the second author brought her perspective as a trainee benefiting from research capacity-strengthening activities, helping to distinguish mechanisms from contexts. As a realist methodologist and having worked in international research partnerships to train (post)graduate trainees, the last author’s experience helped ensure that the analysis process was robust, sound, and grounded in realist philosophy. The reflexivity practice, congruent with the Indigenous realist evaluation (Mutua *et al.* 2025), would help readers situate our perspective and standpoint in the research and identify where our decisions or judgements could be influenced by unconscious bias. Given our different and, to some extent, common positionalities, we regularly discussed the analysis decisions, the participants’ quotes and the theory refinement process among ourselves until a consensus was reached, consistent with Jackson *et al.* (2022) and Teeling *et al.* (2022).

## Results

Two within-case programme theories are presented. Our reporting adheres to the RAMESES II reporting standards for realist evaluation (see Supplementary File S2).

### Programme theory 1: inspire, challenge, eye-opening, empowerment, and sense of agency [case A]

As a research-intensive university (C), trainees and the PI highlighted that the university provides opportunities for students to network with visiting professors/researchers and present their work to them, which can help the students receive feedback from experts. The ARISE programme has allowed the PI and trainees to access multidisciplinary research expertise and equipment (I) that have been pivotal to strengthening the master’s/PhD trainees’ capacity. The multidisciplinary research approach has enabled students to engage with researchers and experts from other disciplines, thereby allowing them to learn from and share knowledge with one another. The self-drive and commitment (C) coupled with the programme resources ignited *‘*a sense of agency’ (M) needed to thrive as a trainee. The participant highlighted that the multidisciplinary nature of the research (C) had empowered them (M) with the technical research skills (O) needed to confront some of the complex research questions. The ‘complementary training resources’ (C) and the ‘multidisciplinary nature of research’ (C) triggered the ‘empowerment’ of technical research skills.

…you can be based here in a research-intensive university that provides all that kind of support (C1) that I’ve just mentioned to you, but if you don’t have the commitment and the self-drive to push through the PhD journey yourself (C2), then you won’t survive in that university. All this additional training and workshop (I)run by the university make a huge difference in our research experience and the increasing demand for multidisciplinary research, and our department is actively pushing researchers to work collaboratively with other social science researchers (C3). …this has been empowering with useful skills and knowledge (M1), and I feel challenged to push the boundaries in my research practice (M2). [Storytelling, PhD trainee 2, Case A]

What makes the difference here is the commitment and the grit, I mean, that spirit of not giving up even when the odds are against you (C2). You are good to go if you have that characteristic and add the support provided by the ARISE and the university. I believe all that sort of enacts *a sense of agency (M3)* required to thrive as a student researcher. …when Dr.[name] was recruiting me to join the ARISE, she asked me some questions to gauge the level of my commitment and sense of self-drive and I think all scholarship or funding providers usually seek to understand that (C2). The multidisciplinary nature of the programme here at [university] [C3] has allowed me and my colleagues to confront some of the questions that a traditional engineer won’t ask. It has *empowered* me (M4) to think of how my innovation project might affect or be perceived by different categories of people. …the multidisciplinary research [C3] simply *empowers you (M4)* to extend the frontiers of knowledge by asking those complex questions [improved technical research skills] [O1]. [Storytelling, PhD Trainee 1, Case A]

The PI and the students highlighted that, as a research-intensive university, the university provided a wide range of complementary training opportunities that helped equip the students with critical technical research and soft skills. Some complementary training has included building researchers’ profiles, research grant writing, PhD research proposal development (for master’s students) and postdoctoral fellowship (for PhD students), research innovation, and entrepreneurship. The complementary university resources and the programme resources have empowered (M) the trainees who have been able to transition from master’s to PhD programmes (O). A collaborator evinced the following:

…when [student] participated in the PhD proposal development workshop that was facilitated by [Name] from the Faculty of Health [complementary training] [C], it empowered her [M4], and she subsequently worked on her application and put in a compelling application. …she has since secured a place in the ARISE project to continue her research work, but this time as a PhD researcher [masters-PhD transition] [O3]. [Interview, Collaborator, Case A]

The trainees shared that given the limited career opportunities in their specialized research field (C), they were not keen to return to Africa shortly. According to the trainees, the opportunities provided by ARISE and their universities were both eye-opening (M) and empowering for them (M), making it possible for them to secure career opportunities in the Global North (O), thus leading to brain drain (O). For instance, some of the trainees deemed the opportunities provided by the programme to present their research work to visiting professors as ‘eye-opening’ because they significantly broadened their perspectives and led to a new realization, as the visiting researchers provided the trainees with feedback. Notably, one of the participants posited that with them securing opportunities in the North, the ARISE programme had yielded undesired outcomes as the graduates ended up working in the Global North, thus depriving the African continent of the critically needed research capacity.

…it worked well that I was already based in a research-intensive university [C1], and I had all this training support and networking opportunities by ARISE and my department. There were multiple times when Dr. [supervisor] told us, as ARISE trainees, to present to the visiting professors and researchers, and such events became useful forums to showcase our research and receive constructive feedback from the experts [C1]. These were quite eye-opening for me [M5], and I had those aha moments, which became a turning point in my research training. …the ARISE equipped me with the skills to fly [empowerment] [M4], and the university released me from the cage. …we have a serious shortage of opportunities in my field in Africa [C4] but, hey, I have secured a job here in Europe [O4], thanks to the skills that the ARISE equipped me with [empowerment] [M4]. [Storytelling, Masters trainee, Case A]

What would you do if you knew you’d struggle to get opportunities in Africa (C4) and an opportunity has opened up in Europe? The first instinct is always to survive, and that’s why I chose survival. …I think the ARISE seeks to build research capacity for researchers dedicated to research in Africa, but now that I’ve reflected on it, I think it has yielded the opposite effect. The capacity benefits Europe, whose capacity is already established [brain drain] [O5]. But it is what it is. [Storytelling, PhD Student, Case A]

In this case, multiple context conditions (complementary training opportunities by the university, demand for multidisciplinary research, and the trainees’ commitment and self-drive for research) were responsible for triggering multiple mechanisms (inspiration, challenge, empowerment, eye-opening experiences, and sense of agency) necessary for generating multiple outcomes (improved technical research skills, research funding secured and master’s to PhD transitions). Notably, as shown in Box 2 below, where such context conditions existed, but the trainees faced limited career opportunities in Africa, the empowerment and eye-opening experiences generated undesired outcomes (trainees securing career opportunities in the Global North, thus depriving the African continent of the much-needed research capacity).

Box 2. **Programme theory 1**

**IF** the trainees are based in a research-intensive university that provides complementary training opportunities (C1) where there is demand for multidisciplinary research (C2), have the commitment and self-drive to undertake academic research (C3), and there are limited career opportunities in their specialized field in Africa (C4) and the ARISE provides research training support, resources and opportunities (I),

**THEN** the trainees’ technical research and soft skills will be improved (O1), additional research funding secured (O2), record master’s and PhD transitions (O3), and secure opportunities in the Global North (O4), thus leading to brain drain (O5),

**BECAUSE** the masters/PhD trainees will be inspired (M1), challenged (M2), their sense of agency activated (M3), empowered (M4), and gain eye-opening experiences (M5).

Given that the Indigenous realist evaluation approach focuses on transformative change, conversations with the ARISE programme partners examined ‘scientific brain drain’ as an undesired outcome in the context of RCS in Africa. Clearly, brain drain deprives the African research ecosystem of talented researchers; an unintended negative outcome of what RCS programmes (like the ARISE) aim to achieve in Africa. In respect of the Indigenous ‘responsibility’ principle, the evaluator initiated a conversation with the ARISE partners about the need to address systemic factors that compel trainees and researchers to seek opportunities outside the African continent. During the conversation, a participant reacted as evinced in the quote below.

…we are very much aware of these challenges, which we've been working on for years. Unfortunately, there seems to be no silver bullet for this challenge because you cannot control people and their desires. However, we have several initiatives targeting African scientists, including [programme], which has very strong cooperation with African researchers and institutions and provides grants to and encourages African researchers working outside Africa to return and re-establish their research activities in their home or African countries. …I know brain-drain is a big problem that clearly cannot be addressed through a single initiative like ARISE and it certainly should be part of policy dialogue with the African governments and other stakeholders. [Interview, Partner C]

Although the partners highlighted that there were no panaceas for the scientific ‘brain drain’ problem, one of the partners pointed out that initiatives targeting African researchers in diaspora were being designed to provide them with research grants and opportunities to return to and (re)establish their research activities in Africa. Partners also highlighted the need for policy dialogues with African governments and other actors on how to protect the African research capacity by preventing ‘brain drain’ from happening in the first place.

### Programme theory 2: inspiration, motivation, commitment, and empowerment [case C]

For ARISE trainees who already hold a [junior] faculty position (C), they reported that they were inspired (M) by their supervisors/colleagues in their university to engage in high-quality research and grow in their career. The trainees also highlighted that their university provided protected time for research (C), allowing them to focus fully on their research without distraction from teaching work, and access to full funding, including stipends (C), and enacted motivation (M) and commitment (M). Notably, the research collaboration with a well-capacitated Global North research partner (C) triggered empowerment (M), as trainees could travel to the Global North partner institution to acquire critical research skills and share knowledge (I) with other students and researchers. The trainees highlighted that all the ARISE trainee support through the PI and the collaborators, and the financial resources to participate in academic workshops and research forums (I) contributed to their empowerment (M). The inspiration, motivation, commitment, and empowerment (M) enabled the trainees to develop a clear research career plan, thus generating high-quality research outputs (O) and advancing their research (faculty) careers (O).

I think I’m well placed, given that I’ve been working as a junior faculty staff [C1] and thus part of the university. …I have been inspired by my senior colleagues [M1] to position myself as a researcher and seize the research opportunities that come my way. So, now that I’m surrounded by colleagues and with the ARISE support towards my PhD, I feel quite motivated [M2] and certainly committed now that I have the financial support needed to undertake my PhD research work [M3]. …the collaboration we have with [Global North University] [C4], which is well established in terms of research infrastructure and expertise, has been empowering for me [M3] as I have been able to travel to Europe and learn from their facility and interact with other students and researchers. I now have some research outputs, including high-quality publications for [O1]. I have also been promoted to a lecturer position, and with a PhD qualification, I will be well-positioned to grow very fast in my research career [O2]. [Storytelling, PhD Student 2, Case C]

…he has been an exemplary student committed to quality research [M3], and he’s been the lead author for two research papers, one published and the other just submitted to a journal [O2]. Being a junior researcher at the university gives him an edge over other PhD students [C1] because he has all the support he can get from colleagues and the ARISE researchers. …the fact that his research is fully funded and he’s drawing a stipend [C2], and of course, the university has given him time off to focus on his PhD studies [C3], so that motivates him even more [M2] to fully focus on his research. [Interview, PI, Case C]

…I think the university giving them protected time for research has made a huge difference [C] because then they have the time to do research. The ARISE’s full financial support [C] can also be a motivator [M] coz they don’t need to worry about their lives or family needs, the small money they get can help. For students affiliated with projects like the ARISE, this ensures they are motivated [M3] and committed to their research course [M4]. [Interview, Collaborator, Case C]

Since the ARISE seeks to strengthen research capacity for African researchers who are dedicated to research on the continent, this Case shows that supporting [junior] faculty staff in African universities and providing them with the favourable environment (protected time for research and full research funding) (C) can empower (M) them to grow in their career and within the universities—see Box 3 below. Notably, collaboration with Global North research partners can allow the trainees to leverage the partner’s research equipment and expertise (I) not available in the host institution, thus empowering (M) them and enabling them to generate high**-**quality research outputs (O). Inspired by the Indigenous ‘decolonization’ principle which seeks to explore, understand and challenge colonial power structures and dynamics, the relationships between the ARISE partners, the PIs and their global North and South collaborators were explored to establish who had control over what resources and how that influenced the implementation of the initiative and the extent to which the relationships were equitable (Mutua *et al.* 2025). The Bottom-Up approach to the ARISE programme design guaranteed the PIs the autonomy and freedom to establish partnerships or collaborations with partners from anywhere and who could add value to their research project, such as providing complementary research equipment and expertise, as highlighted by some of the trainees who reported benefiting from such resources made available by their global North partner (Mutua *et al.* 2025).

Box 3. **Programme theory 2**

**IF** the trainee is a junior faculty staff (C1), has fully funded research (C2) and their university provides protected time for PhD research (C3), and the ARISE provides training support, resources and opportunities (I),

**THEN** the trainees will increase their research outputs (O1) and record a growth in their faculty/research careers (O2),

**BECAUSE** they will be inspired (M1), motivated (M2), committed (M3), and empowered to undertake high-quality research and grow in research career (M4).

### Overarching programme theory

**Figure 2.**
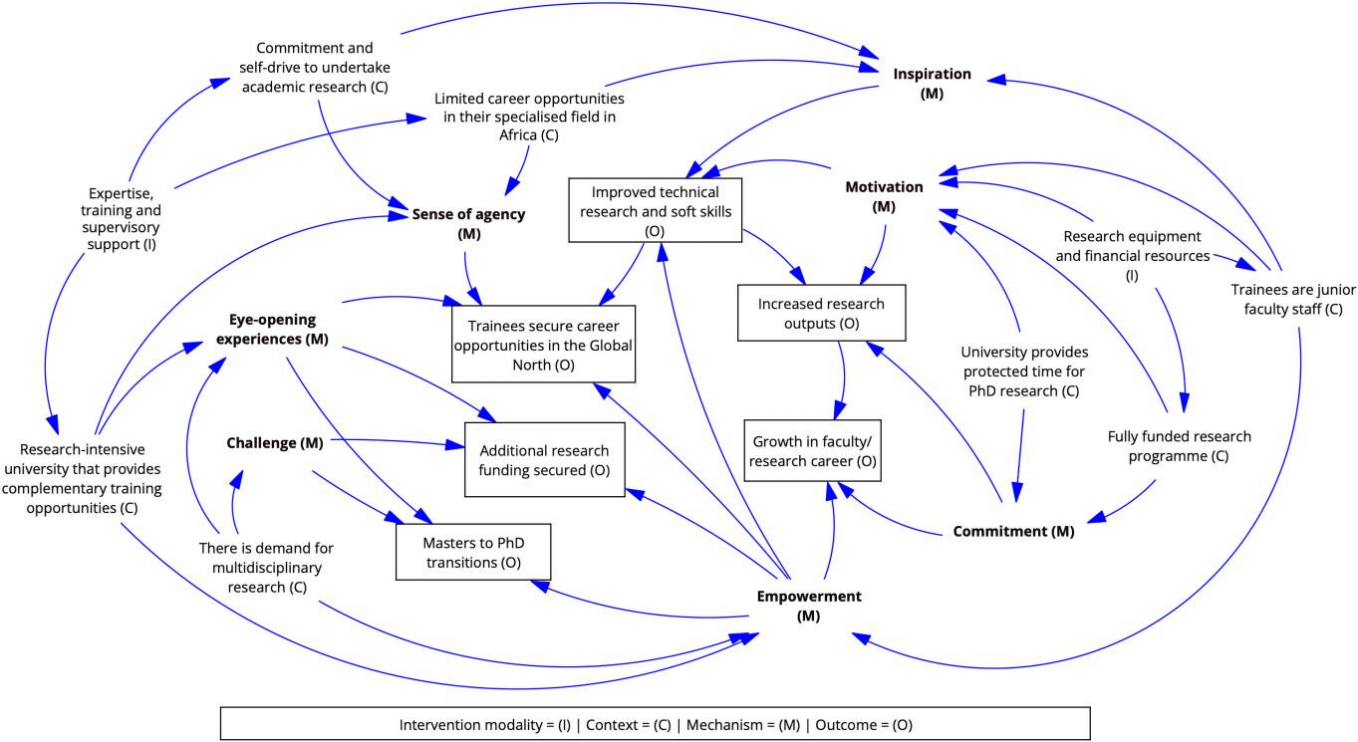
Overarching programme theory.

Figure 2 represents a causal loop diagram, which illustrates the theory generated after the dispositional analysis to retroduce a theory explaining how the ARISE research partnership strengthens the health research capacities of the (post)graduate trainees across the two university contexts.

Our refined programme theory captures those mechanisms that are triggered when the ARISE intervention components are introduced into different university contexts. For completeness, it captures the mechanisms that yield positive outcomes (motivation, sense of agency, commitment, inspiration and eye-opening experiences). It is evident, based on Fig. 2, that strengthening research capacity for postgraduate trainees is a complex endeavour as multiple contextual conditions are required for one or more mechanisms necessary for generating intended or unintended research capacity outcomes to be triggered. Based on the study evidence, various elements of the IPT have been validated, modified and refined.

First, ‘Availability of supervisory capacity’ and formation of ‘intra-Africa research collaborations’ (framed as contexts in the IPT) were deemed as part of the programme architecture. As such, they were modified to intervention (I) elements in the refined theory. For instance, the supervisory capacity is factored into the programme architecture as each PI is required to commit their time and leverage their collaborators’ resources and expertise to support at least two PhD trainees over the ARISE project period. Similarly, the need for intra-African research collaborations as context (in the IPT) is refined because research collaborations and networks are already guaranteed within the ARISE architecture. As in Case A, transdisciplinary research is possible through collaboration, which allows trainees to access varied expertise and research support, challenging, inspiring, and providing them with eye-opening experiences. Notably, having ‘adequate government funding to support the training of master’s/PhD students’ (framed as a context in the IPT) was not validated because the ARISE programme itself provides the funding/resources.

Second, the selection of the postgraduate trainees is also critical in a RCS initiative. Targeting individuals who already possess the commitment and self-motivation to undertake academic research ensures that they have the necessary soft skills and values to complete their postgraduate training. With the support of training and supervision, committed and self-driven trainees can acquire the necessary tools (technical research and soft skills), which may further enable them to secure additional research funding and transition from a master’s to a PhD programme.

Third, the transition into seasoned health researchers in Africa is contingent on the broader research ecosystem, such as the availability of career opportunities in specialized fields in Africa. Therefore, unexpected outcomes such as brain drain are possible. Although ‘transition into seasoned health researchers in Africa’ was not identified as an outcome, some trainees argued that they were on a trajectory to becoming seasoned researchers, thus it cannot be refuted as a potential outcome. This is because the study captured shorter-term outcomes, rather than medium- or long-term outcomes (Mutua 2025).

Lastly, Case C programme theory is an emergent or new theory, distinct from the IPT. The theory focuses on postgraduate trainees who are also junior faculty staff in an African university. Contrary to Case A, the provision for protected time for PhD research by the university is crucial for trainees in Case C, as it enables them to focus more on their PhD work without being overwhelmed by teaching and administrative responsibilities. The training support, resources, and opportunities provided by the RCS programme will inspire, motivate, and empower trainees in a context where they are afforded protected time by the university and financial support for research. As a result, the trainees can generate more research outputs (publications) and grow in their university faculty careers through promotion.

## Discussion

This study has established that international research partnerships work differently across contexts to strengthen the health research capacity of (post)graduate trainees. While supervisory capacity and establishment of intra-Africa research collaborations and availability of government funding for training were framed (in the IPT) as contextual conditions necessary for triggering perceived support among trainees, our study has established that the two cases are characterized by different contextual conditions that influence the mechanism activated. This paper describes the varied mechanisms (i.e. inspiration, challenge, sense of agency, empowerment, eye-opening experiences, motivation, and commitment) that were triggered across the two university contexts to generate desirable outcomes (i.e. improved technical research and soft skills, additional research funding secured, masters to PhD transitions, increased research outputs and growth in faculty research career recorded) and undesirable outcome (i.e. trainees secure career opportunities in the Global North thus depriving the African continent of the much-needed research capacity).

While multiple challenges affect the postgraduate trainees such as limited funding, lack of access to research infrastructure and expertise and dysfunctional support systems for trainees (Jowi 2021), this study has established that international research partnerships help mitigate some of these challenges. International research partnerships provide trainees access to financial support, research expertise, and infrastructure not available at their host university and career-defining networking opportunities. This study has established that access to critical research equipment, expertise, training, and supervisory support empowers, inspires, challenges, and activates the trainees’ sense of agency and provides them with eye-opening opportunities, specifically in contexts characterized by multidisciplinary research. The trainees become committed and driven to undertake academic research. Our findings show that selecting the right trainees is pivotal in ensuring that the programme’s support and resources yield improved research capacity outcomes, as the trainee needs to be suitably motivated and committed to undertaking independent research consistent with Polkinghorne *et al.* (2023).

Bates *et al.* (2011) highlight the lack of systematic skills development in postgraduate training as a critical gap. Our study has established that the provision of training support and opportunities by the Case A host university (which is a research-intensive institution) ensures that the trainees acquire the requisite skills in grant writing, journal publication, proposal development for PhD and postdoctoral applicants, research innovation and entrepreneurship which are needed to thrive in a research career. Case A institution was termed a research-intensive university given its strong research culture, international research funding base and research outputs (Mutua and Mukumbang 2025). Owing to attending grants writing and PhD proposal development workshops, some of the ARISE trainees reported writing successful grant applications, securing additional research funding, and transitioning from master’s to PhD programmes. The training and supervisory support provided to the trainees improved their research capacity by challenging and empowering them, inspiring and exposing them to eye-opening experiences. It is not surprising that when trainees gain practical and tangible skills in academic writing and preparing grant proposals, improvements in scientific presentations, publications, and the acquisition of research grants can be observed (Merry *et al.* 2019, Cassell *et al.* 2022, Balogun *et al.* 2024). On this basis, Garcia-Morante *et al.* (2024) call for a cultural shift in postgraduate training that considers broader competencies beyond the technical research skills, e.g. research communication and writing, innovation, and entrepreneurship skills, which align with career opportunities.

This study has established that the (post)graduate trainees will likely produce improved research outputs (e.g. research articles) in the context where the curricula include writing skills and there is commitment and motivation to undertake a PhD by the trainee (Raffing *et al.* 2017). As demonstrated in the results (see ‘Programme theory 2’ section), through the ARISE research partnership, the trainees were empowered, inspired, committed, and motivated, particularly because of the effective training support, resources, and opportunities provided by the ARISE, which allowed them to increase their research outputs and record career growth. The provision of protected time for research specifically contributes to a positive postgraduate research environment, culture, and development opportunities (Knight *et al.* 2024). In addition, providing the trainees with stipends motivates them to focus fully on their PhD research, making it possible to generate high-quality research outputs (Ali *et al.* 2017). As junior faculty staff, the (post)graduate trainees will likely be inspired by their supervisors (colleagues), motivated to undertake quality research, and consequently generate research outputs. On this basis, Ndejjo *et al.* (2022) highlight that when a postgraduate trainee has a clear [potential] career pathway and is positioned for it early, it can produce a transformative PhD student on a path to success. In this Case, the junior faculty can benefit from the formal training and the ‘hidden curriculum’, including supervision, role models, research collaboration, networking, and research exchanges (Tokalić 2023) made possible through the ARISE support and resources. No wonder the networking and collaboration opportunities provided by international research partnerships have intangible benefits to the postgraduates as they enhance motivation and career prospects, according to El Hajj *et al.* (2024). Notably, being junior faculty staff, the trainees have their supervisors as colleagues, and this can—beyond triggering inspiration (mechanism)—mitigate the power imbalance between the supervisor and student (Polkinghorne *et al.* 2023). This ensures that the supervisor-trainee relationship is not defined as a ‘leader–follower’ relationship, which can be problematic if it is not well managed (Vähämäki *et al.* 2021).

While ARISE contributes to strengthening the health research capacity in Africa, some trainees have moved to the Global North countries (brain drain), thus depriving the African continent of the much-needed health research capacity. McAlpine *et al.* (2023) have acknowledged that the mobility of postgraduate trainees is a complex problem, especially as the trainees must negotiate (as agents) their intentions and long-term career goals. Although this study established that the partners were aware of the systemic challenges affecting RCS efforts, including the brain drain problem, acknowledging the complexity of the challenge highlights the need to approach RCS from a systems perspective. We argue that if the health research capacity is to benefit the African institutions and wider society, measures need to be put in place to improve the research and innovation environment and provide opportunities for young, talented people to retain their talent on the continent. Specifically, national governments will need to take ownership, invest financial resources in research and development, and enhance the research and innovation ecosystem (Wiyeh and Mukumbang 2025), which will require collaborative initiatives involving academic institutions, government bodies, and international partners (Omoya *et al.* 2025).

The Bottom-Up approach employed in designing the ARISE partnership was critical in ensuring the voices of African stakeholders were central to defining the research challenges and priorities, as well as how to address them, considering their local contexts (Mutua *et al.* 2025). Abimbola *et al.* (2024) argue that the top-down approaches, where the researchers and policymakers ‘prescribe’ solutions, are more common, and they render the interpretations at the periphery (by African actors) about the challenges (African research ecosystem) not central to shaping the understanding, analysis or solutions to the challenges. We argue that it is possible for a well-intentioned and equitable international research partnership like the ARISE (Mutua *et al.* 2025), seeking to strengthen capacities for postgraduate trainees to yield unintended outcomes like brain drain because the wider research system does not enable the African researchers and research to thrive. Arguably, this could be a flaw in most international research partnerships that seek to strengthen individual-level capacity and develop a critical mass of researchers in Africa, without addressing systemic challenges that render the research environment or ecosystem unconducive. Perry *et al.* (2022) posit that ‘wicked problems don’t have linear ﬁxes; the best toolkit in the world cannot predict or settle the uncertainties, whims, and unpredictability within the complexity of intra-acting systems of humans, environments, and institutions’ (p. 643). To this end we contend that addressing research capacity gaps in the African ecosystem is a typical case of a wicked problem and requires a comprehensive approach to addressing it.

Fundamentally, based on the evidence shared in this paper, the use of international research partnerships to deliver (post)graduate training and supervision can yield transformative results for the trainees by strengthening their research capacity. However, for such efforts to effectively yield positive research capacity outcomes, there is a need to understand the complexity that characterizes those efforts, including the research environment/ecosystem, the trainee’s traits and motivations, and the knowledge and expertise of the supervisory team (Everitt 2025). By drawing on the programme and research partnership resources, the postgraduate trainees can overcome limitations in their own institution’s systems and facilities by accessing a wide range of international expertise and support from collaborators and researchers affiliated with the research partnership/programme mentors (El Hajj *et al.* 2024). While being embedded within a research project supported through an international research partnership may provide the trainees access to a wide range of research infrastructure, expertise, and support, the provision of complementary training resources, and opportunities by the host university/institution can optimize the impact of the partnership.

The Indigenous lens to the realist evaluation approach was valuable in multiple ways. The Indigenous lens enabled the exploration and understanding of the subject (master’s and PhD students) in LMIC contexts as well as the colonial ideologies and power dynamics characteristic of the ARISE partnerships and collaborations (Mutua *et al.* 2025). Notably, the Indigenous ‘reverence’ principle, which emphasizes the need to recognize the value of spirituality as central to knowledge contribution, did not play out in the study, potentially because of the intellectualized environment (i.e. a university setting rather than a typical Indigenous community setting) within which the initiative is implemented. In line with the relationality principle, the voices of diverse participants (e.g. PIs, collaborators, students, partners) were central to the CMO configuration process following triangulation of evidence. The research guaranteed the participants’ rights to data ownership by giving them the opportunity to review their accounts (transcripts) and edit them, and their involvement in the validation and dissemination workshops where the study findings were shared and next course of action discussed, in line with the ‘rights and regulations’ and ‘respectful representation’ principles. In line with the ‘responsibility’, the evaluator highlighted to the ARISE stakeholders/participants the need to focus on strengthening research systems and consider the uniqueness of each implementation context in delivering the RCS intervention to prevent unintended outcomes (e.g. brain drain) from occurring. In respect of the ‘responsivity’ principle, the evaluator was able to adapt the interview questions across the two cases, thereby probing and meaningfully interrogating the various contexts and mechanisms that generate specific outcomes in each context. Additionally, the ‘reciprocity’ principle (which emphasizes the need to identify the actual value added to the participants’ lives by an intervention), inspired the exploration of positive and negative, intended and unintended outcomes as reported in this paper. Lastly, the ‘decolonization’ principle inspired a deep interrogation of the nature of the ARISE partnership and collaborations among the PIs and their global South and North partners and they were broadly positioned to deliver research capacity outcomes (Mutua *et al.* 2025). In respect of the ‘decolonization’ principle, our research concluded that it is the equitable nature of the ARISE partnership that guaranteed African PIs autonomy and liberty to choose collaborators and establish meaningful relationships, owing to their control over the programme resources (Mutua *et al.* 2025).

This paper is one of the few studies that have engaged with the complexity of international research partnerships to understand how and why they contribute to strengthening the research capacities of postgraduate trainees in African universities. The evidence presented in this paper is relevant to policy and programme practitioners as it can inform how they design, implement and evaluate similar interventions in similar contexts. The key limitation of the study relates to the inclusion of just two cases, the small number of participants across the two cases and the use of qualitative methods only. Lack of application of quantitative methods meant that the research capacity outcomes patterns could not be quantified. The choice of qualitative methods only was based on the small number of cases and programme recipients across the two cases. Future studies should encompass as diverse a range of university contexts as possible and quantitatively explore research capacity outcomes patterns for postgraduate trainees.

## Conclusion

Our paper establishes that an international research partnership works differently to strengthen health research capacity for (post)graduate trainees across African university contexts. International research partnerships that provide access to a wide range of expertise, equipment, training, and supervisory support to (post)graduate trainees will result in improved technical and soft skills, increased research outputs, securing of additional research funding, transition from master’s to PhD, securing job opportunities and recording a research/faculty career growth. This is because the trainees will gain eye-opening experiences, be inspired, challenged, and empowered, and their sense of agency will be activated. Trainees who are already [junior] faculty staff in a university that provides protected research time are in an advantageous position because they can quickly leverage their technical and soft skills thus resulting in increased research outputs (e.g. publications) and growth in their faculty careers. This is because the trainees will be inspired, motivated and empowered through the RCS support, resources, and opportunities. We conclude that research capacity strengthening initiatives targeting (post)graduate students need to consider ‘inter alia’ the university contexts (e.g. complementary resources and opportunities provided, provisions of protected research time for junior faculty), the individual traits and readiness for (post)graduate training (motivation and commitment to independent PhD research) and the likelihood of the trainees’ capacity benefiting the African research ecosystem.

## Supplementary Material

czaf055_Supplementary_Data

## Data Availability

All relevant data have been included within the paper.

## Reflexivity statement

The authors include one female and two males. While two of the authors are early-career researchers from the Global South, the third is a senior researcher/academic in the Global North. The third author is mentoring the two early-career authors. One author is a specialist in evaluation and health research systems, while the other two have a speciality in health systems and policy research. All three authors have varied experience conducting realist evaluations in multiple African countries.
