# Supplementary material for: Strengthening health research capacity for postgraduate trainees: an indigenous realist evaluation of the ‘African Research Initiative for Scientific Excellence’ programme
Source: Health Policy Plan. 2025 Aug 20;40(9):967–80. doi: 10.1093/heapol/czaf055 (PMC12516029; doi:10.1093/heapol/czaf055)
Supplement: czaf055_Supplementary_Data [file czaf055_supplementary_data.zip › Supplemental file 1.docx]

***CMO codes***

| **Step** | **CMO elements extracted**  [*The number (in parentheses) represents the number of documents or transcripts from which the C, M or O element was extracted*] |
| --- | --- |
| Step 1: ARISE document review | Sufficient supervisory capacity [C] (3)  Host institution has adequate research and training resources for masters/PhD students [C] (2)  Individual research capacities strengthened [O] (1) |
| Step 2: FGDs and interviews | Trainees have financial support and access to a wide range of research expertise and equipment [C] (3)  PI has sufficient supervisory capacity [C] (2)  Based in a research institution that has adequate resources/ government funding to support the training of masters/PhD students [C] (3)  Acquire the right knowledge and skills [O] (3)  Perceived support [M] (2)  Transition into seasoned health researchers in Africa [O] (5) |

The CMO elements extracted from the ARISE documents (Step 1) were used to hypothesise that:

IF *the PI has sufficient supervisory capacity (C1) and the host institution has adequate research and training resources for master’s/PhD students (C2), THEN the trainees’ readiness for health-related careers* would be *improved* (O), BECAUSE *the training, supervision and mentorship would equip the students with the right skills (M).*

Following the FGDs with project-level stakeholders and interviews with the programme partners, respectively, the draft IPT was revised as shown in the Table below:

***IPT Modification Process***

| **Draft IPT** | **Modification** | **Revised IPT** |
| --- | --- | --- |
| IF the PI has sufficient supervisory capacity (C1) and the host institution has adequate research and training resources for masters/PhD students (C2), THEN the trainees’ readiness for health-related careers would be improved (O), BECAUSE the training, supervision and mentorship would equip the students with the right skills (M). | The intervention (I) “the trainees have financial support and access to a wide range of research expertise and equipment” added to the IPT  Mechanism modified to ‘acquire the right knowledge and skills’ and ‘perceived support’  Outcome modified to ‘masters/PhD trainees will transition into seasoned health researchers in Africa’ | IF the PI has sufficient supervisory capacity (C1) and are based in a research institution that has adequate resources to support the training of masters/PhD students (C2) and the trainees have financial support and access to a wide range of research expertise and equipment (I), THEN the masters/PhD trainees will transition into seasoned health researchers in Africa (O), BECAUSE they will acquire the right knowledge and perceived support (M). |
