# Supplementary figures and images for: Strengthening health research capacity for postgraduate trainees: an indigenous realist evaluation of the ‘African Research Initiative for Scientific Excellence’ programme
Source: Health Policy Plan. 2025 Aug 20;40(9):967–80. doi: 10.1093/heapol/czaf055 (PMC12516029; doi:10.1093/heapol/czaf055)

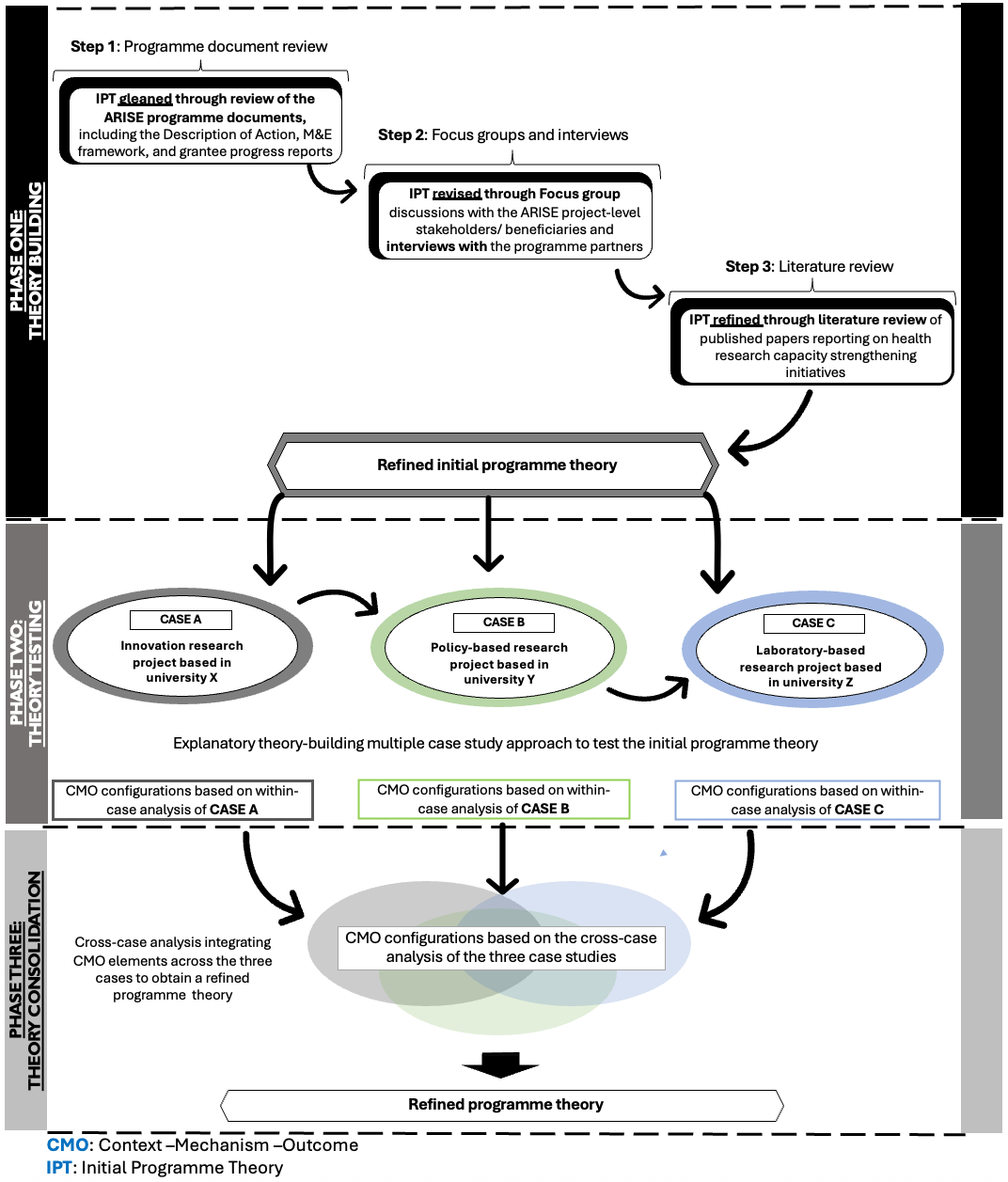

Supplement: czaf055_Supplementary_Data [file czaf055_supplementary_data.zip › Supplemental figure 1.tiff]
